# The Effects of Triterpenoid Saponins from the Seeds of *Momordica cochinchinensis* on Adipocyte Differentiation and Mature Adipocyte Inflammation

**DOI:** 10.3390/plants9080984

**Published:** 2020-08-03

**Authors:** Jae Sik Yu, Namood E. Sahar, Yan-Ran Bi, Kiwon Jung, Changhyun Pang, Joo Young Huh, Ki Hyun Kim

**Affiliations:** 1 School of Pharmacy, Sungkyunkwan University, Suwon 16419, Korea; jsyu@bu.edu; 2 College of Pharmacy, Chonnam National University, Gwangju 61186, Korea; sahar.namood@gmail.com (N.E.S.); bibi1994623@gmail.com (Y.-R.B.); 3 Institute of Pharmaceutical Sciences, College of Pharmacy, CHA University, Seongnam 13488, Korea; pharmj@cha.ac.kr; 4 School of Chemical Engineering, Sungkyunkwan University, Suwon 16419, Korea; chpang@skku.edu

**Keywords:** Saponin, *Momordica cochinchinensis*, obesity, diabetes, adipocyte

## Abstract

Obesity is a medical condition in which abnormal or excessive fat accumulates to an extent that is associated with various diseases. In our ongoing research to figure out natural products with anti-obesity effects, a phytochemical investigation of the EtOH extract of the seeds of *Momordica cochinchinensis* was carried out, which resulted in the isolation of two major triterpenoid saponins: gypsogenin 3-*O*-*β*-d-galactopyranosyl(1→2)-[*α*-l-rhamnopyranosyl (1→3)]-*β*-d-glucuronopyranoside (**1**) and quillaic acid 3-*O*-*β*-d-galactopyranosyl(1→2)-[*α*-l-rhamnopyranosyl(1→3)]-*β*-d-glucuronopyranoside (**2**). Then, the effects of the isolated triterpenoid saponins (**1** and **2**) on adipocyte differentiation were evaluated, and it was demonstrated that the isolated saponin (**1**) showed inhibitory effects on adipogenesis. In mature adipocytes, the isolated saponin (**1**) reversed tumor necrosis factor α (TNFα)-induced proinflammatory cytokine gene expression. Additionally, the isolated saponin (**1**) reduced lipolytic gene expression leading to decreased basal lipolysis activity. Collectively, these findings suggest that saponin (**1**) of *M. cochinchinensis* exerts beneficial effects in the regulation of adipogenesis and adipocyte inflammation and could be a potential therapeutic alternative in the treatment of obesity-induced metabolic diseases.

## 1. Introduction

Obesity is an epidemic, with over two billon people worldwide being either overweight or obese [1]. In obese individuals, dysfunctional adipose tissue and the saturation of its expanding capacity leads to lipid overflow [2]. This overflow leads to lipid accumulation in peripheral organs, which is involved in the pathophysiology of various diseases, including metabolic diseases, cardiovascular diseases, and cancer [3]. In particular, the accumulation of excessive fat in the adipose tissue results in the development of inflammation and oxidative stress in adipocytes leading to impaired adipocyte insulin resistance and overall metabolic dysregulation [4,5]. Therefore, the maintenance of adipocyte health is important for the regulation of metabolism at the systemic level.

During the development of obesity, the adipose tissue expands via increases in the size (hypertrophy) and number (hyperplasia) of adipocytes [6], with both processes being considered targets for anti-obesity drugs. Obese individuals present both hypertrophy and hyperplasia of fat cells, whereas only hypertrophy is observed in overweight individuals, compared to lean individuals, suggesting that adipogenesis plays a key role in the development of obesity [7]. An increase in adipocyte number through differentiation is a complex process that involves various transcriptional factors, such as fatty acid binding protein 4 (FABP4), CCAAT/enhancer binding protein α (C/EBPα), and peroxisome proliferator-activated receptor (PPARγ) [8]. In addition, the expansion of adipose tissue in obesity is accompanied by the infiltration of immune cells and secretion of proinflammatory cytokines. As a result, the adipocytes remain under a low-grade chronic inflammation state, which further aggravates their metabolic dysregulation [9,10].

As part of our ongoing studies to identify bioactive natural products from diverse natural sources [11,12,13,14,15], we conducted a phytochemical investigation of the seeds of *Momordica cochinchinensis* Spreng. (Cucurbitaceae). *M. cochinchinensis* is indigenous to south Asia and is distributed throughout the region, from southern China to northern Australia. In south Asia, the plant is popularly known as gac in Vietnamese and as spiny bitter gourd, baby jackfruit, and red melon in other regions. Gac is known to have various physiological benefits, and it is considered a nutraceutical in southeast Asia [16]. The red aril surrounding the seeds of the fruit is consumed as food, and a fully ripe fruit is known to be an excellent source of lycopene and β-carotene, efficient in preventing vitamin A deficiency [17,18]. The seeds of *M. cochinchinensis* are well-known as Momordicae Semen in traditional Chinese medicine. These dried seeds are used for a variety of therapeutic purposes, such as rheumatic pain, hemorrhoids, hemangiomas, wounds, bruises, and muscular spasms [19]. A previous phytochemical investigation of these seeds for secondary metabolites showed that it contains a variety of triterpenoids and saponins [20]. Related reports also demonstrated that the constituents of Momordicae Semen showed anti-inflammatory, antioxidant, and anti-gastritis effects [21]. In a recent study, our chemical investigations of the seeds of *M. cochinchinensis* resulted in the purification of two major saponins that showed anti-proliferative activity in human lung cancer cell lines (A549, H1299, Calu-6, and H1264) and attenuated primary lung endothelial cell proliferation [22]. In this study, a phytochemical analysis of the EtOH extract of the seeds of *M. cochinchinensis* led to the isolation of two triterpenoid saponins (**1** and **2**); their structures were elucidated based on spectroscopic data of NMR and LC/MS analysis. Additionally, the effects of these triterpenoid saponins on adipocyte differentiation and mature adipocyte metabolism were examined.

## 2. Materials and Methods

### 2.1. General Experimental Procedures

Optical rotations were obtained using a JASCO DIP-1000 digital polarimeter (Jasco, Tokyo, Japan). IR spectra were determined using a JASCO FT/IR 300E spectrophotometer (Jasco, Tokyo, Japan). Nuclear magnetic resonance (NMR) spectra, including the distortionless enhancement by polarization transfer (DEPT), heteronuclear single quantum correlation (HSQC), heteronuclear multiple bond correlation (HMBC), total correlation spectroscopy (TOCSY), and nuclear overhauser effect spectroscopy (NOESY) experiments, were obtained using a Bruker AVANCE III 700 NMR spectrometer operating at 700 MHz (^1^H) and 175 MHz (^13^C) (Bruker), with chemical shifts given in ppm. Preparative high-performance liquid chromatography (HPLC) was performed using a Waters 1525 Binary HPLC pump with a Waters 996 Photodiode Array Detector (Waters Corporation, Milford, CT, USA). Semi-preparative HPLC was conducted using a Shimadzu Prominence HPLC System with SPD-20A/20AV Series Prominence HPLC UV-Vis Detectors (Shimadzu, Tokyo, Japan). LC/MS analysis was performed on an Agilent 1200 Series HPLC system, equipped with a diode array detector and 6130 Series ESI mass spectrometer. An analytical Kinetex C18 100 Å column (100 × 2.1 mm, 5 μm; flow rate: 0.3 mL/min; Phenomenex) was used. Furthermore, silica gel 60 (Merck, 230–400 mesh) and RP-C18 silica gel (Merck, 40–63 μm) were used for column chromatography. Merck pre-coated Silica gel F254 plates and RP-18 F254s plates (Merck, Darmstadt, Germany) were used for thin-layer chromatography (TLC). Spots obtained after TLC were detected under ultraviolet (UV) light or by heating after spraying with anisaldehyde-sulfuric acid.

### 2.2. Plant Material

The seeds of *M. cochinchinensis* were collected in May 2010 from a local medicinal herb store in Yunnan province, People’s Republic of China, and identified by one of the authors (K.H. Kim). A voucher sample (MBJ-2010-05) was deposited in the herbarium of the School of Pharmacy, Sungkyunkwan University, Suwon, Korea.

### 2.3. Extraction and Isolation

The kernels (2.4 kg) of the *M. cochinchinensis* seeds were powdered and extracted with 50% ethanol (EtOH) under reflux twice for 4 h each. After filtering and concentrating in vacuo, the resultant residue (123 g) was suspended in deionized water and then partitioned with hexane, ethyl acetate (EtOAc), and *n*-BuOH, yielding 6.3, 9.5, and 34.8 g, respectively. Based on the yield of the three fractions obtained, the *n*-BuOH soluble fraction was considered as the main fraction and was subjected to further isolation. A partial amount of the *n*-BuOH soluble fraction (10 g) was separated using preparative HPLC (Eclipse DBX-C18 column, Agilent, 250 mm × 21.2 mm i.d., 7 μm, flow rate of 5 mL/min) using a gradient solvent system from 10% MeOH to 80% MeOH for 90 min, which yielded seven fractions (B1–B7). Among these subfractions, fraction B7 (450 mg) was identified to have significant compounds based on LC/MS analysis; it was subsequently purified by semi-preparative reversed phase HPLC using a Luna phenyl-hexyl column (250 mm × 10 mm inner diameter, 10 μm) with 30% MeCN (flow rate: 2 mL/min) to yield compounds **1** (60 mg) and **2** (85 mg).

### 2.4. Cell Culture

3T3-L1 cells (American Type Culture Collection, Rockville, MD, USA) were cultured as previously described [23]. Briefly, the preadipocytes were cultured in Dulbecco’s modified Eagle’s medium (DMEM; Hyclone, Logan, UT, USA) supplemented with 10% fetal bovine serum (FBS; Hyclone) and 1% penicillin/streptomycin and incubated at 37 °C in a 5% CO_2_ incubator. Two days after the 3T3-L1 preadipocytes reached 100% confluency (day 0), the cells were treated with differentiation media 1 (5 µg/mL insulin, 0.25 mM dexamethasone, and 0.5 mM 1-methyl-3-isobutylxanthine). On day 2, the cells were exposed to differentiation media 2 (5 μg/mL insulin). From day 4 to 8, the medium was replaced every 48 h with fresh 10% FBS DMEM media (differentiation media 3). The cells were treated with the isolates on day 0 and day 2 to examine the effects of these saponins on adipogenesis. To observe the compounds’ effects on mature adipocytes, the cells were pretreated with 100 μM of the isolated compounds for 1 h before treatment with 10 ng/mL tumor necrosis factor α (TNFα).

### 2.5. Cell Viability

Cell viability was determined using a 3-(4,5-dimethylthiazol-2-yl)-2,5-diphenyl-tetrazolium-bromide (MTT) assay [24,25,26]. The 3T3-L1 preadipocytes were seeded in 96 wells and grown until 70–80% confluence. Then, the cells were treated with various concentrations of compounds **1** and **2** for 24 h. At the end of the incubation period, 10 µL of the MTT reagent (final concentration 0.5 mg/mL) was added to each well for 2 h at 37 °C in a 5% CO_2_ incubator. Then, media was removed and 200 µL DMSO was added for 10 min before measuring the absorbance at 540 nm.

### 2.6. Oil Red O (ORO) Staining

On day 8, the cells were washed with PBS and fixed with 10% formalin for 1 h. Oil Red O solution was prepared by diluting 30 mL of 0.5% Oil Red O stock solution (Sigma, Saint Louis, MO, USA) with 20 mL of distilled water. Fixed cells were stained with the working solution for 1 h and were washed with PBS. After representative pictures were taken, the stained cells were dissolved with 100% isopropanol for quantification at 490 nm.

### 2.7. Western Blotting

On day 4, the cells were harvested and lysed in RIPA buffer (Thermo Scientific, Rockford, IL, USA), separated by SDS-PAGE, and transferred to PVDF membranes. The membranes were incubated with primary antibodies (Cell Signaling, Danvers, MA, USA; FABP4, PPARγ, and C/EBPα) overnight. The blots were detected using a LAS-3000 Luminescent Image Analyzer (Fuji photo film, Tokyo, Japan).

### 2.8. Gene Expression Analysis

Total RNA was extracted using TRI Reagent (TR118, MRC, Cincinnati, OH, USA). cDNA was synthesized using TOPscript™ RT DryMIX (Enzynomics, Daejeon, Korea). mRNA levels were measured via real-time PCR using Rotor-Gene Q (QIAGEN, Chatsworth, CA, USA) with a 20-μL reaction volume consisting of cDNA transcripts, primer pairs, and a TOPreal SYBR Green PCR Kit (Enzynomics, Daejeon, Korea). The gene expression levels were normalized to 18S rRNA levels.

### 2.9. Lipolysis Assay

On day 8, after differentiation, compound **1** was administered to the 3T3-L1 adipocytes. After 24 h, the cells were washed and incubated with a lipolysis assay buffer for 2 h. The released glycerol content in the medium was measured using a lipolysis colorimetric assay kit (Sigma, Saint Louis, MO, USA). Absorbance was measured at 570 nm.

### 2.10. Statistical Analysis

All statistical analyses were performed using the StatView software. Mean values obtained from each group were compared by ANOVA. All results are expressed as the mean ± SEM. A *p*-value < 0.05 was used as the criterion for a statistically significant difference.

## 3. Results and Discussion

### 3.1. Isolation and Structural Identification of Compounds

**Figure 1:**
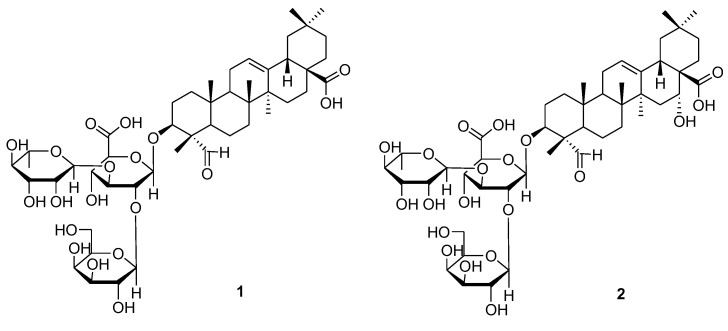
Chemical structures of the triterpenoid saponins (**1** and **2**).

The EtOH extract of the *M. cochinchinensis* seeds was employed to fractionation of three fractions using hexane, EtOAc, and *n*-BuOH. By a comprehensive process of LC/MS analysis, the *n*-BuOH soluble fraction was chosen to undergo a chemical analysis, which resulted in the isolation, by HPLC, and identification of two triterpenoid saponins. The isolated compounds **1** and **2** were structurally elucidated by analysis of spectroscopic data, including ^1^H and ^13^C NMR and specific rotation as well as LC/MS analysis. The compounds were characterized to be gypsogenin.3-*O*-*β*-d-galactopyranosyl(1→2)-[*α*-l-rhamnopyranosyl(1→3)]-*β*-d-glucuronopyranoside (1) [22] and quillaic acid 3-*O*-*β*-d-galactopyranosyl(1→2)-[*α*-l-rhamnopyranosyl(1→3)]-*β*-d-glucuronopyranoside (2) [22] (Figure 1).

### 3.2. The Effects of the Isolated Saponins on Adipocyte Differentiation

**Figure 2:**
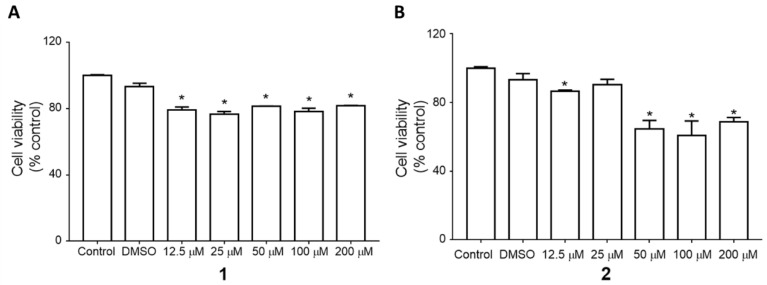
Cytotoxicity of compounds **1** and **2**. Cell viability was examined in 3T3-L1 preadipocytes treated with various doses (12.5–200 µM) of compounds **1** (**A**) and **2** (**B**) for 24 h. The values are the means ± SEM of three experiments. * *p* < 0.05 vs. control (no treatment).

First, the effects of the isolated saponins (**1** and **2**) on cell viability were tested. Compounds **1** and **2** were administered at various doses for 24 h to the 3T3-L1 preadipocytes (Figure 2). Compound **1** reduced the cell viability to approximately 80% compared to that in the control, up to 200 µM. In contrast, compound **2** presented the highest cell toxicity as shown by the reduced cell viability (~60%) at 50 µM. Thus, we selected compound **1** for the subsequent experiments.

**Figure 3:**
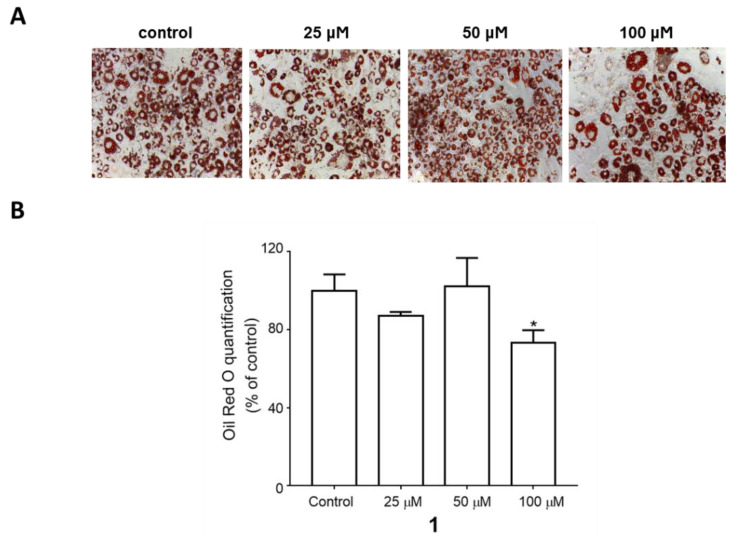
The inhibitory effect of compound **1** on lipid accumulation during adipocyte differentiation. 3T3-L1 preadipocytes were treated with various concentrations of compound **1** from the start of adipocyte differentiation (day 0) until day 4. (**A**) The representative Oil Red O staining pictures of differentiated adipocytes on day 8. (**B**) Quantification of Oil Red O staining. The values are the means ± SEM of three experiments. * *p* < 0.05 vs. control.

**Figure 4:**
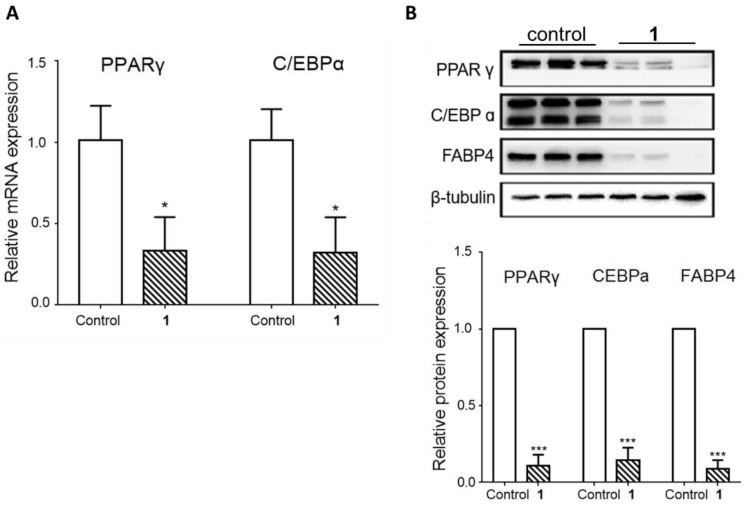
Effects of compound **1** on markers of adipocyte differentiation. 3T3-L1 preadipocytes were treated with 100 µM of compound **1** from the start of adipocyte differentiation (day 0) until the 4th day. (**A**) Gene expression of the adipogenic markers C/EBPα and PPARγ on day 4 of differentiation. (**B**) Representative image and quantification of C/EBPα, PPARγ, and FABP4 protein expression on day 4 of differentiation. The values are the means ± SEM of three experiments. * *p* < 0.05 vs. control, *** *p* < 0.001 vs. control.

To examine the effects of triterpenoid saponin (**1**) on adipocyte differentiation, compound **1** was administered to the cells from the beginning of the differentiation process until the fourth day, which is the critical period for adipogenesis. On day 8, Oil Red O staining was performed to visualize the mature adipocytes, as indicated in Figure 3 by the accumulation of lipids shown in red. The result showed that compound **1** significantly inhibited lipid accumulation at the 100 µM concentration (Figure 3A). The quantification of Oil Red O staining confirmed the inhibitory effect of compound **1** against adipocyte differentiation (Figure 3B). To verify this effect, the gene and protein levels of adipogenic markers were measured on day 4 during differentiation. As shown in Figure 4, the gene expression of C/EBPα and PPARγ and protein expression of C/EBPα, PPARγ, and FABP4 were significantly inhibited upon compound **1** treatment. Other saponins from natural products have been previously reported to exert inhibitory effects on adipocyte differentiation. Notably, ginseng whole extracts and various ginsenosides have been shown to suppress adipogenesis [27]. For example, ginsenoside Rc was reported to efficiently inhibit adipogenesis in 3T3-L1 adipocytes, which was mediated by the downregulated expression of the major adipogenic transcription activator (PPAR-γ and C/EBP-α) proteins of the adipogenesis pathway [28]. Recently, ginsenoside Rg2, one of the specific ginsenosides in red ginseng, was demonstrated to decrease the expression levels of adipogenic transcription factors [PPARγ, C/EBPα, and sterol regulatory element binding protein 1c (SREBP1-c)] and regulate target genes such as acetyl-CoA carboxylase (ACC) and fatty acid synthase (FAS), as well as significantly promote AMP-activated protein kinase (AMPK) [29]. In addition, the anti-obesity effects of ginsenoside Rg1 in 3T3-L1 adipocyte cells and high-fat diet-induced obese C57BL/6J mice were reported, where ginsenoside Rg1 showed an anti-adipogenic effect via down-regulating the mRNA expression of C/EBPα, PPARγ, and SREBP-1c, adipogenic transcription factors, and activated the AMPK pathway [30]. Similarly, our data confirmed that triterpenoid saponin (**1**) inhibits the adipogenic process through the regulation of adipogenic transcription factors.

### 3.3. The Effects of Triterpenoid Saponin (***1***) on Mature Adipocyte Metabolism

**Figure 5:**
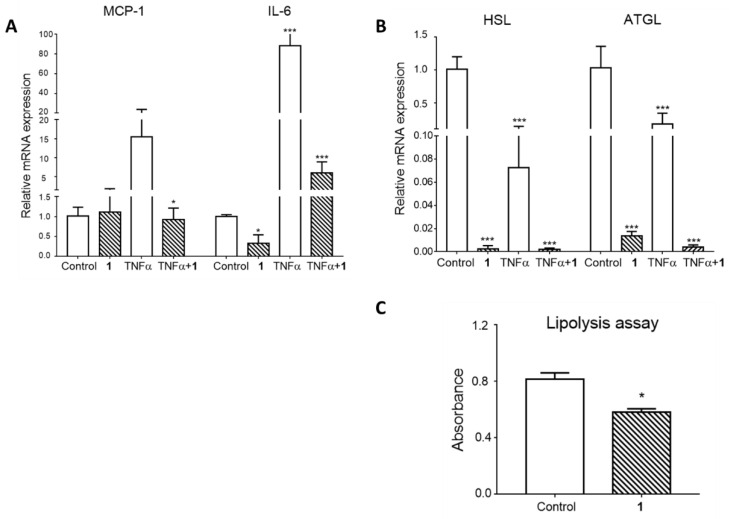
Effects of compound **1** on mature adipocyte gene expression and lipolysis. (**A**,**B**) 3T3-L1 mature adipocytes were pretreated with 100 µM of compound **1** for 1 h, followed by 10 ng/mL tumor necrosis factor α (TNFα) treatment for 6 h. Gene expression was measured using real-time PCR. (**C**) After 24-h treatment with or without compound **1**, lipolysis was measured according to the glycerol content in the incubation medium. The values are the means ± SEM of three experiments. * *p* < 0.05 vs. control, *** *p* < 0.001 vs. control.

Along with the effect of triterpenoid saponin (**1**) on adipogenesis, its effects on mature adipocyte metabolism were also examined. Our group and others have previously reported that saponins isolated from *M. cochinchinensis* seeds have anti-inflammatory activities through the inhibition of the expression of nitric oxide and suppression of inflammatory signaling molecules such as NF-κB in macrophages [23,31]. Proinflammatory signaling is one of the critical factors in the development of adipocyte insulin resistance [9,10]. Tumor necrosis factor alpha (TNFα) is a proinflammatory cytokine that is detected at high levels in individuals that are obese as well as in those with type 2 diabetes [32,33]. Additionally, hypertrophied adipocytes are known to exhibit increased TNFα expression, which negatively regulates insulin signaling [34,35]. To simulate metabolic dysregulation in fully differentiated adipocytes, mature cells were treated with TNFα for 24 h. To observe whether saponin (**1**) could protect adipocytes from the TNFα negative effects, one of the experimental conditions included 1-h pretreatment with compound **1**. Firstly, the gene expression of various proinflammatory cytokines was measured. As expected, TNFα led to a significant increase in the mRNA levels of MCP-1 and IL-6. Interestingly, pretreatment with compound **1** reversed the TNFα-induced overexpression of MCP-1 and IL-6 (Figure 5A).

In an obese state, an increase in the basal lipolysis rate in adipocytes leads to the hydrolysis of stored triglycerides, which causes the release of free fatty acids (FFAs). The increase in the circulating FFA level results in lipid accumulation in peripheral tissues, such as muscle and liver, and, ultimately, the development of insulin resistance and metabolic syndrome [36,37]. Therefore, adipose tissue lipolysis is considered the main target of anti-obesity drug development. Nicotinic acid is an example of a lipid-lowering agent that acts through the inhibition of adipose tissue lipolysis [38]. In line with this notion, the gene expression of the lipolysis enzymes adipose triglyceride lipase (ATGL) and hormone-sensitive lipase (HSL) was examined. As a result, TNFα significantly downregulated the mRNA expression of both HSL and ATGL. Moreover, treatment with compound **1** dramatically suppressed the gene expression of HSL and ATGL at the basal level and in the TNFα-treated condition (Figure 5B). To determine whether the reduced gene expression is related to adipocyte function, the lipolysis assay was conducted. As shown in Figure 5C, treatment with compound **1** significantly inhibited lipolysis compared to the control, which is in line with the reduced mRNA expression results. Collectively, these results imply that triterpenoid saponin (**1**) ameliorates adipocyte inflammation and suppresses lipolysis in mature adipocytes.

## 4. Conclusions

Here, we described the chemical analysis of the EtOH extract of the seeds of *M. cochinchinensis* and the isolation of two triterpenoid saponins (**1** and **2**). Our study demonstrates that the triterpenoid saponin, gypsogenin 3-*O*-*β*-d-galactopyranosyl (1→2)-[*α*-l-rhamnopyranosyl (1→3)]-*β*-d-glucuronopyranoside (**1**) exerts anti-obesity effects through the inhibition of adipogenesis and mature adipocyte inflammation and lipolysis. To date, most data regarding the pharmacological role of *M. cochinchinensis* has been focused on its anticancer activities [39]. In this study, the administration of compound **1** to preadipocytes resulted in the suppression of the gene and protein expression of adipogenic transcription factors, which led to the inhibition of adipocyte differentiation, as shown by the reduced lipid accumulation. Moreover, the treatment of the mature adipocytes with compound **1** resulted in reductions in the levels of TNFα-induced proinflammatory cytokines and basal lipolysis. To the best of our knowledge, this is the first study to determine the effects of the seeds of *M. cochinchinensis* on adipocytes. Our findings provide experimental evidence of the therapeutic potential of compound **1** for the treatment of obesity-induced metabolic diseases, which should be further assessed in animal models.
